# Hospital Wastewater—Important Source of Multidrug Resistant Coliform Bacteria with ESBL-Production

**DOI:** 10.3390/ijerph17217827

**Published:** 2020-10-26

**Authors:** Kristína Lépesová, Petra Olejníková, Tomáš Mackuľak, Klára Cverenkárová, Monika Krahulcová, Lucia Bírošová

**Affiliations:** 1 Faculty of Chemical and Food Technology, Department of Nutrition and Food Quality Assessment, Slovak University of Technology, Radlinského 9, 81237 Bratislava, Slovakia; nagyovak5@gmail.com (K.L.); klara.cverenkarova@stuba.sk (K.C.); monika.krahulcova@stuba.sk (M.K.); 2 Faculty of Chemical and Food Technology, Institute of Biochemistry and Microbiology, Slovak University of Technology, Radlinského 9, 81237 Bratislava, Slovakia; petra.olejnikova@stuba.sk; 3 Department of Environmental Engineering, Faculty of Chemical and Food Technology, Slovak University of Technology, Radlinského 9, 81237 Bratislava, Slovakia; tomas.mackulak@stuba.sk

**Keywords:** antibiotic resistance, hospital wastewaters, ESBL, biofilm, efflux pumps

## Abstract

This work compares the prevalence of antibiotic resistant coliform bacteria in hospital wastewater effluents in Slovak (SR) and Czech Republic (ČR). It also describes selected antibiotic resistant isolates in view of resistance mechanism and virulence factor. The highest number of multidrug resistant bacteria was detected in samples from the hospital in Valašské Meziříčí (ČR). More than half of resistant isolates showed multidrug resistance phenotype as well as strong ability to form biofilm. In 42% of isolates efflux pump overproduction was detected together with *tetA* and *tetE* genes. The production of extended-spectrum β-lactamases in coliform isolates was encoded mainly by *bla_TEM_*, *bla_CTX-M-2_* and *bla_CTX-M-8/25_* genes. About 62% of resistants contained a combination of two or more extended spectrum beta-lactamases (ESBL) genes. Our results strengthen the fact that hospital effluents are a source of multidrug resistant bacteria which can spread their resistance genes to other bacteria in wastewater treatment plants (WWTPs). Accordingly, hospital wastewater should be better treated before it enters urban sewerage.

## 1. Introduction

Recently, the World Health Organization announced that antibiotic resistance is emerging, and we are fast running out of treatment options [1]. In Europe, 20–30% of hospitalized patients receive antibiotics (ATB) treatment during their hospitalization. This creates good conditions for the development of ATB resistance due to high selective pressure. In addition, pathogenic bacteria can also spread in the hospital through the health care personnel. Antibiotic resistant bacteria (ARB) may escape from these facilities in infected patients and the sewer system. This situation may be worsened when effluents from healthcare facilities are directly discharged with no prior treatment in the wastewater network. Sewerage systems collect wastewater not only from households but also from hospitals, psychiatric clinics, retirement homes and other health care facilities that are a major source of pharmaceuticals [2]. Hospital effluents (HEs) are ranked as a special category because of their highly hazardous and toxic character. These types of wastewater contain cocktail of ATB, disinfectants, metabolized drugs, and sensitive and resistant bacteria from hospitalized patients [3]. ATBs in subinhibitory concentrations presented in wastewater act as signal molecules and regulatory substances inducing horizontal gene transfer, mutagenesis, and SOS bacterial response as well as the selection of resistant bacterial strains [4]. This makes HEs significant and important contributors to the spread and development of ATB resistance in wastewater and the environment. Some studies have reported lower levels of ARB and ATB resistance genes (ARGs) in municipal wastewater compared to HEs [5,6,7]. This means that wastewater released from healthcare facilities can increase the number of coliforms inflowing to the municipal WWTPs. According to this fact, HEs should be better treated before entering sewerage. This treatment could involve the use of boron doped diamond electrodes, ferrates or ozonation [8,9].

In 2017 the World Health Organization published a list of ATB-resistant “priority pathogens”. The most critical group of all includes multidrug resistant (MDR) bacteria that pose a particular threat in hospitals, nursing homes, and among patients whose care requires devices such as ventilators and blood catheters. These include *Acinetobacter* spp., *Pseudomonas* spp. and various *Enterobacteriaceae* (including *Klebsiella* spp., *E. coli*, *Serratia* spp., and *Proteus* spp.) producing extended spectrum beta-lactamases (ESBL) or carbapenemases [10]. ESBL-producing bacteria cause resistance to β-lactam ATBs containing an oxyimino group (e.g., ceftazidime, cefotaxime, aztreonam) together with resistance to other classes of non-penicillin ATBs, including fluoroquinolones (FQ), aminoglycosides, trimethoprim/sulfa-methoxazole and ß-lactam/ß-lactamase inhibitor combinations. These bacteria are responsible for host prolonged hospital stay, increased treatment costs, morbidity, and mortality [11]. Recent studies have shown the high frequency of human intestinal carriage of ESBL-producing *E. coli* in both hospital and community settings [5]. In Paris a 10-fold increase in the rate of healthy subjects with ESBL-producing *E. coli* fecal carriage over a five-year period (2006–2011) was found [11]. Between 2002 and 2013 the incidence of ESBL-producing *Enterobacteriaceae* increased up to four-fold in French hospitals. During this decade, the proportion of *E. coli* producing ESBL increased among all strains of *Enterobacteriaceae* family from 19–59% [5]. 

Biofilm formation is one of the most important virulence factors of pathogenic bacteria, which enable bacteria to escape the host defense mechanism. Moreover, it was shown that bacteria in biofilm are 1000 times more resistant due to slow drugs penetration and altered microenvironment [12]. Additionally, a biofilm matrix can improve intracellular communication as well as horizontal gene transfer due to physical proximity and cell density, while the conjugation can be up to 700 times more effective compared to wild-type bacterial cells [13]. Sabir et al. (2017) highlighted the increased incidence of urinary tract infections caused by *E. coli*, *E. cloacae* or *K. pneumoniae* showing a strong intensity of biofilm production which can subsequently be released into wastewater [12]. 

This study compares the occurrence of ATB-resistant coliform bacteria in effluents from hospitals and healthcare facilities. The second part of the work is focused on the identification and characterization of selected ATB-resistant coliform bacteria isolated from HEs.

## 2. Materials and Methods

### 2.1. Characterization of Healthcare Facilities and Description of Sampling Methodology

Samples of wastewater were collected at the outlet point of canalization in healthcare facilities situated in the Slovak and Czech Republics, as 2 h-decanted samples in sterile falcon tubes between 2014 and 2017. Two different samples were collected from each facility. The HE samples were immediately transferred into the laboratory for microbiology analysis. The data regarding the number of beds, equipment type and recipient of wastewater are listed in Table 1. Most listed hospitals treat wastewater only by nitrification. Wastewater from the National Cancer Institute of St. Elizabeth (NCISE), the University Hospital in Ružinov, the Hospital in Malacky, the Children’s Faculty Hospital and the University Hospital of Academician Ladislav Dérer is chlorinated and nitrificated before connecting to the public sewerage network. 

### 2.2. The Occurrence of ATB-Resistant Coliform Bacteria in HEs

For coliform bacteria and *E. coli* enumeration in wastewater samples collected from healthcare facilities, a diagnostic medium-Chromocult Coliform agar (VWR Chemicals, USA) stated by ISO 9308 (2014) was used [14]. Samples of HEs were diluted and subsequently inoculated on ATB agar plates with the same procedure as in the case of influent wastewaters described in the study of Lépesová et al. (2018) [15]. Ampicillin (AMP), gentamicin (GEN), ciprofloxacin (CIP) and chloramphenicol (CMP) resistance of coliform bacteria was tested in concentrations defined by the European Committee on Antimicrobial Susceptibility Testing (EUCAST) for the European Union [16] as well as by the Clinical Laboratory Standards Institute (CLSI) for the United States [17]. Tetracycline (TET)-resistance was studied only according to the CLSI since EUCAST does not provide resistance breakpoints [17].

All experiments were performed in tree parallels and were repeated three times. Cultivation conditions of coliform bacteria were 37 °C, 24 h. 

### 2.3. Isolation and Identification of Resistant Coliform Bacteria

Coliform bacteria showing resistance to applied ATBs were isolated by streak plate method on Mueller Hinton agar (MH) (Biolife, Italy) and incubated at 37 °C for 24 h. After obtaining separate colonies of pure cultures, identification via a Matrix–Assisted Laser Desorption/Ionization–Time of Flight (MALDI–TOF) mass spectrometer according to Lépesová et al. (2018) was performed [15]. Briefly, fresh bacterial culture was spotted onto a steel target plate (Bruker Daltonics Inc., Billerica, MA) and dried at room temperature. The MALDI-TOF α-cyano-4-hydroxycinnamic acid matrix was prepared daily as a saturated solution in 50% acetonitrile and 2.5% trifluoroacetic acid (TFA). The sample to be analyzed was consequently spotted with 1 μL of matrix solution. Samples were evaluated by use of an AutoFlex I TOF-TOF apparatus (Bruker Daltonics Inc., Billerica, MA) in linear positive-ion mode across the m/z range of 2000 to 20,000 with gating of ions below m/z 400 and a delayed extraction time of 450 ns. Each spot was measured by using 2000 laser shots at 25 Hz in groups of 50 shots per sampling area of the spot. The data sampling rate was 0.5 GHz. Each plate was calibrated by using a Bacterial Test Standard (Bruker Daltonics). Spectra were analyzed by using MALDI BioTyper software (v 2.0) (BioTyper Library v 3.0; Bruker Daltonics), a proprietary algorithm for spectral pattern matching resulting in a logarithmic score from 0 to 3. Previous work using discrete bacterial colonies determined that a score of >1.9 indicates species identification, a score of 1.7 to 1.9 indicates genus identification, and a score of <1.7 indicated no identification.

### 2.4. ATB Susceptibility Detection 

#### 2.4.1. Macro-Dilution Method Assay

In resistant coliform bacteria, except *E. coli* and *Klebsiella* spp., isolated from hospital wastewaters, the susceptibility to various ATBs including AMP, ceftazidim (CAZ), meropenem (MER), GEN, CIP, CMP and TET was detected by a plate dilution drop method according to Lépesová et al. (2018) [15]. However, *E.coli* and *Klebsiella* spp. are frequent and typical producers of ESBLs and carbapenemases, and for these strains wider antibiotic spectrum was applied (see Section 2.4.2).

#### 2.4.2. Microtiter Plate Assay

In case of isolates identified as *E. coli* and *Klebsiella* spp. a commercial microtiter plate assay for resistance profile detection was used. During the method, the manufacturer´s manual was followed. MicrolatestMIC test (Erba Lachema, Czech Republic) contains 24 ATBs divided into two plates G I and G II for each tested bacterial isolate. While the plate G I detects resistance to AMP, ampicillin/sulbactam (AMS), aztreonam (AZT), amikacin (AMK), GEN, CIP, CMP, colistin (COL), trimethoprim/sulfamethoxazole (T/S), TET, cefazolin (CFZ) and cefuroxime (CXM), plate G II serves to determine the sensitivity of isolates to piperacillin (PIP), piperacillin/tazobactam (PIT), tobramycin (TOB), netilmicin (NET), tigecycline (TGC), CAZ, cefotaxime (CTX), cefoperazone (CPZ), cefoperazone/sulbactam (CPS), cefepime (CEP), ertapenem (ERT) and MER. Microtiter plates with inoculum were incubated statically at 37 °C for 16–20 h and the growth of bacterial cultures was evaluated spectrophotometrically (λ = 630 nm) using a plate reader (BioTek, US). The results were assessed according to the manufacturer´s manual.

### 2.5. Double Disk Synergy Test for the Production of ESBLs

Inoculum (2 mL) of 24 h bacterial culture in physiological saline was prepared to a final density of 0.5 McFarland scale. For each tested isolate, a pair of MH agar plates with and without oxacillin (c = 128 mg/L) (Sigma Aldrich, Germany) was prepared. Agar plates were inoculated with prepared bacterial suspensions via sterile cotton sticks on which the ATB disks of amoxicillin/clavulanic acid (AMC), AZT, CAZ, CTX and CEP (Oxoid, UK) were applied according to the scheme published in the study of Hrabák et al. (2008) [18]. After cultivation for 24 h at 37 °C the growth of inhibitory zones around the applied ATB disks was observed.

### 2.6. Ethidium Bromide (EtBr) Agar Cartwheel Method for Efflux Pumps Overproduction

Overnight cultures (16–18 h) of ATB-resistant coliform isolates in MH broth were prepared. The bacterial suspension density was again adjusted to 0.5 McFarland in physiological saline and MH agar plates with EtBr (Serva, Germany) at a concentration 2.5 mg.L^−1^ were prepared. Each culture of resistant coliform bacteria was seeded onto the surface of EtBr agar plates with a sterile cotton stick. Incubation of agar plates took place for 16–18 h at 37 °C and the activity of the efflux pumps was subsequently detected after UV irradiation [19].

### 2.7. Biofilm Detection Assay

The method for biofilm detection has been previously described in the study of Lépesová et al. (2018) [15]. A quantitative assessment of biofilm formation was detected by measuring the absorbance of biofilm eluate at 570 nm using a plate reader (BioTek, US). 

Based on the absorbance values measured on a spectrophotometer the tested isolates were included among weak, medium, strong, and very strong producers of bacterial biofilm according to the scale developed by Taniguchi et al. (2009) [20]. The scale value intervals were previously described in the study of Lépesová et al. (2018) [15]. *Pseudomonas aeruginosa* (CCM 3955) from the Czech Collection of Bacterial Strains in Brno served as a positive control for the detection of biofilm formation ability by resistant isolates of coliform bacteria. The experiment run in six parallels and was repeated three times.

### 2.8. Antibiotic Resistance Gene Detection 

#### 2.8.1. Multiplex PCR Assay for ESBL Gene Detection

The reaction mixture for multiplex PCR was prepared by mixing of primers (Metabion International AG, Germany) (Table 2) and deionized sterile water for molecular purposes (5 Prime, Germany) in a final volume of 25 μL. Other reagents such as the mix of the deoxyribonucleotide triphosphates (dNTPs), PCR buffer and the Hot Start Taq DNA Polymerase with MgCl_2_ were added to the reaction mixture as a commercial Multiplex Master Mix (MPMX) (Biotechrabbit, Germany) preparation. One colony of each resistant coliform isolate was suspended in the reaction mixture before initial denaturation (94 °C). MPMX in a total volume of 25 μL was added after the initial denaturation and incubation to 72 °C to each sample.

#### 2.8.2. Single PCR Assay for ESBL Gene Detection

The reaction mixture for single PCR was prepared in a final volume of 50 μL by mixing of 5 μL of 10 × concentrated buffer solution of MgCl_2_ for Taq polymerase (100 mmol·L^−1^, Tris/HCl, pH 9 pri 25 °C, 500 mmol·L^−1^ KCl a 1% Triton X-100), 1 μL of 0.2 mmol·L^−1^ dNTPs, 42 μL of deionized sterile water for molecular purposes, 50–100 ng of genomic DNA and defined volume of each primers according to Table 2. As in case of MPMX, 1 U Taq DNA polymerase at a volume of 0.5 μL was added to the reaction mixture after the initial denaturation (94 °C).

#### 2.8.3. PCR Conditions 

PCR was performed under the following conditions: initial denaturation (94 °C/20 min), denaturation (94 °C/40 s), annealing (55 °C for Multiplex I reaction and *bla_CTX-M-15_*, 60 °C/1 min for Multiplex II reaction and *bla_CTX-M-8/25_*), extension (72 °C/1 min) and final extension (72 °C/10 min) in 35 cycles in a thermocycler (Mastercycler personal, Eppendorf, Germany). The size of PCR products was detected by 1.5% (*w*/*v*) agarose gel electrophoresis.

#### 2.8.4. Multiplex PCR Assay for Tet Genes Detection

The multiplex PCR reaction mixture for detection of *tetA* and *tetE* genes encoding the overproduction of tetracycline efflux pumps was prepared as described above. However, each of the primers was applied at a volume of 0.5 μL (Table 2). PCR was performed under the same conditions as in the case of ESBL gene detection with an annealing temperature of 55 °C. In the last step the PCR products were visualized by gel electrophoresis (1.5% (*w*/*v*) agarose gel).

## 3. Results and Discussion

### 3.1. The occurrence of ATB-Resistant Coliform Bacteria in Wastewaters from Healthcare Facilities

Studies show that conventional wastewater treatment renders limited results in terms of ATB and ATB-resistant bacteria and gene removal. Additionally, it might even increase the concentration of certain ATB resistance genes in wastewater which flow further into the environment [24,25,26,27,28]. Due to these facts, the quantification of ATB-resistant bacteria in 18 wastewater samples collected from 10 hospitals and healthcare facilities was performed (Table 1). Monitoring was focused on fecal indicator bacteria in HEs because some representatives of these genera are characterized by MDR and associated with life-threatening infections [1]. 

The total number of coliform bacteria ranged up to 7.18 log CFU·mL^−1^ of which 4.49 log CFU·mL^−1^ was represented by *E. coli* (Table 3). In our previous work the number of coliform bacteria in influent wastewater from three Slovak and three Czech WWTPs ranged from 3.02 to 4.94 log CFU·mL^−1^ [29]. The number of coliform bacteria in some samples of HE is much higher, so this strengthens the fact that HE can significantly contribute to the spread of different bacteria.

Resistance of coliform bacteria was detected against five types of ATBs of different classes including β-lactams, aminoglycosides, FQs, amphenicols and tetracyclines. AMP as β-lactam ATB was selected due to frequent prescription in Slovakia. Both GEN (aminoglycoside) and CIP (FQ) may be present in higher levels due to their frequent use in the treatment of infections caused by Gram-negative bacteria in the community as well as in the hospital sector [30]. The use of CMP is currently limited, but bacterial strains with resistance to this ATB persist due to the possible mechanisms of cross-resistance [31].

In variable samples of HEs different numbers of resistant coliforms were detected. In general, the highest resistance rate was noticed against AMP, which relates to its frequent use in both the Slovak as well as the Czech Republic [30]. Additionally, according to Magiorakos et al. (2012) many strains included in the group of coliform bacteria are equipped with intrinsic resistance to this ATB [32]. In the case of *E. coli* AMP resistance is acquired. However, AMP is rapidly degraded during the wastewater treatment and the prevalence of AMP-resistant bacteria in wastewaters is related to the horizontal transfer of genes encoding the production of β-lactamases [31]. 

Similarly, Le et al. (2016) [3] found in HEs high prevalence of coliform bacteria with MDR phenotype. Effluents from the NCISE and the HVM contained a high number of coliform bacteria showing resistance to all tested ATBs. Although, the number of beds for hospitalized patients is comparable in both hospitals (Table 1), the number of ATB-resistant coliforms was on average two logarithmic orders higher in the HVM. However, it is important to note that wastewater from the NCISE is pre-treated before reaching the public sewerage network (Table 1). The high number of AMP, GEN and CIP resistant coliform bacteria was also observed in effluent samples taken from specialized palliative Hospice Citadela as well as from the Psychiatric Clinic (PC) in Oščadnica. Within the group of coliform bacteria, MDR *E. coli* were detected in the effluent of the NCISE, the PC and in two Czech hospitals (HVM and Vsetín).

### 3.2. Identification and Susceptibility of ATB-Resistant Coliform Bacteria Isolates

Nowadays, a steady increase in the number of ATB-resistant bacteria as well as ARGs complicating the treatment of both human and animal pathogens, not only in the field of clinical medicine but also in native isolates originating from the contaminated aqueous environment, was monitored [33,34]. Thirty-five strains of ATB-resistant coliform bacteria were isolated from two sample HEs—NCISE (18 strains) and PC in Oščadnica (17 strains). The majority was identified as *E. coli* (54%) followed by *Lelliottia amnigena* (17%), *Citrobacter freundii* (14%) and *Enterobacter cloacae* (6%). The remaining isolates were included in the genus of *Citrobacter* (*C. gillenii*—3%) and *Klebsiella* (*K. variicola*—3%, *K. pneumoniae*—3%).

When assessing the ATB susceptibility profile of isolates, the intrinsic AMP, AMS and CFZ resistance of *C. freundii* and *E. cloacae* as well as the intrinsic AMP resistance of *Klebsiella* spp. was considered [32]. Table 4 summarizes the ATB susceptibility profile of isolates.

More than 50% of *E. coli* isolates were resistant to 11 tested ATBs (Table 4). A high rate of resistance to the penicillin-type of ATB (AMP, PIP) as well as to their combination with β-lactam inhibitors was also observed (Table 4). Isolates from the NCISE showed CTX, CAZ, CEP, CFZ and CXM resistance. In *E. coli* isolated from the PC only CFZ and CAZ resistance was observed. Conte et al. (2017) have also isolated MDR bacterial strains identified mainly as *E. coli*, *K. pneumoniae* and *K. oxytoca* from hospital wastewaters in Brazil showing high resistance to CTX as well as CAZ [35]. Resistance to these two antibiotics predicts ESBL production typical for bacteria present in HEs [36]. The increasing MDR bacteria occurrence (especially in hospitals) leads to frequent use of last-choice ATBs such as carbapenems or COL resulting in the development of resistance [30]. Although, resistance of *E. coli* to MER was not detected, 16% of *E.coli* isolates were resistant to ERT and COL. ATB resistant *K. pneumoniae* is the second most dangerous pathogen associated with severe and long-term infections leading to high morbidity and mortality [37]. According to the EARS-Net reports in 2016, up to one third of *K. pneumoniae* isolates were resistant to at least one of the controlled groups of ATBs including FQs, third generation cephalosporins, aminoglycosides and carbapenems. The combined resistance to multiple ATBs was also routinely described [30] as has been shown in the case of an isolate of *K. pneumoniae* that was resistant to COL, NET, TOB as well as to AZT, CAZ, CTX and CEP.

Hospital wastewater offers an environment suitable for the various antibiotic resistance genes to exchange [3]. These facts are also highlighted by results obtained in this study according to which up to 70% of coliforms isolated from the HEs were MDR (Table 4). 

### 3.3. Selected Mechanisms of ATB Resistance in Isolates

#### 3.3.1. Detection of Efflux Pumps Overproduction

Efflux pumps overproduction is the most common reason for the cross-resistance to different ATB [31]. A significant number of *E. coli* isolated from both HEs showed overproduction of efflux pumps (74%) together with the MDR phenotype (86%). Half of the isolates identified as *L. amnigena* and *C. freundii* as well as the isolate *K. variicola* were also characterized by increased efflux (Table 5).

Several studies show that *tetA* and *tetE* genes encoding TET efflux pumps are frequently found in all coliform bacteria, especially in *E. coli* [38,39,40,41]. The presence of *tet* genes was monitored in all isolates (Table 5). The majority of isolates was characterized by the presence of the *tetA* gene, while half of these isolates contained also *tetE* gene. In 13 isolates of *E. coli* the presence of *tetA* gene was detected. Although Chopra and Roberts (2001) noted the presence of the *tetE* gene in particular with *tetB*, *tetI*, *tetC* and *tetD* genes, similarly to Adesoji et al. (2015) the coexistence of this gene with *tetA* in 5 *E. coli* isolates was observed [39,42]. All of the isolates identified as *C. freundii*, *L. amnigena*, *K. pneumoniae* and *K. variicola* acquired *tetA* gene. Four isolates of *C. freundii* as well as one isolate of *L. amnigena* and *K. variicola* contained this gene, encoding the increased production of efflux pumps (Table 5).

#### 3.3.2. Detection of ESBL Production

Phenotype of ESBL production was observed in seven isolates identified as *E. coli* as well as in four isolates of *C. freundii*, two isolates of *L. amnigena* and one isolate of *E. cloacae* (Table 5). Similarly, Yamashita et al. (2017) detected a high prevalence of ESBL-producing strains of *E. coli* in hospital wastewaters and subsequently in the rivers of Japan [43]. The production of the AmpC type of β-lactamases is also clinically important because their over-expression provides resistance to a wide range of drugs including penicillin, cephalosporins, cefoxitin and inhibitors of β-lactamases. According to the EUCAST, the most common AmpC producers are *E. coli*, *K. pneumoniae*, *K. oxytoca* and *Salmonella enterica* [44]. AmpC enzyme production was detected only in one isolate of *E. coli* from the effluent taken in the NCISE. As a result of mutations in constitutive genes a hyperproduction of chromosomally encoded AmpC β-lactamases may occur, especially in strains of *Enterobacter* spp., *Citrobacter* spp., *Serratia* spp. and *Acinetobacter* spp. [45]. Hyperproduction of AmpC in combination with ESBL production was detected in one isolate of *L. amnigena* from the effluent of the NCISE (Table 5).

Gram-negative bacteria bearing multiple *bla* genes (e.g., *bla_NDM_*, *bla_KPC_*, *bla_CTX-M_*, and *bla_SHV_*) are increasingly found in hospital wastewaters, together with other common and related ARGs such as *qnr* (FQ), *erm* (macrolide), *sul* (sulfonamides), and *tet* (TET) [3]. The production of ESBLs encoded by *bla_TEM_*, *bla_SHV_* or *bla_CTX-M_* genes are considered as most frequent among Enterobacteriaceae over the last decade [21,22]. Thirty isolates resistant to AMP and cephalosporin ATBs were screened for the presence of *bla_TEM_*, *bla_OXA_* and *bla_SHV_* genes. TEM-type β-lactamases are characteristic for *E. coli* isolates, while SHV enzymes predominate in *Klebsiella* spp. [46]. Nevertheless, in case of *E. coli* isolates the presence of the TEM (9 isolates) and SHV (8 isolates) group of genes was quite similar and the frequency of the occurrence of *bla_OXA_* genes was much lower (two isolates) (Table 5). Another study pointed out the significant presence of both *bla_TEM_* and *bla_SHV_* genes in ESBL-producing *E. coli* isolated from patients with urinary tract infection [47]. All of these results suggest the presence of high numbers of ARB harboring *bla_TEM_* genes in hospital environments. A significant proportion (70%) of *E. coli* isolated from the effluent of NCISE possessed a group of *bla_SHV_* genes in the absence of genes encoding TEM-type ESBLs. In the study of Haller et al. (2018) 33 of 94 Gram-negative isolates from HEs in Singapore were also positive for the presence of *bla_SHV_* genes [48]. In all isolates of *C. freundii*, *C. gillenii* as well as *K. variicola* and *K. pneumoniae*, the presence of genes encoding a group of TEM and OXA β-lactamases was observed. In accordance with Zhu et al. (2017) *bla_SHV_* genes occurred in all isolates of *Klebsiella* spp. (Table 5) [46]. 

Unlike other ESBLs, the CTX-M family constitutes a complex and non-homogeneous group of enzymes. Nowadays, among all types of ESBLs, CTX-M enzymes are much more widespread in *Enterobacteriaceae* not only in Europe but also in other parts of the world [21]. In all tested isolates of *C. freundii* (five isolates) and *K. variicola* (one isolate) from the effluent of the PC in Oščadnica, the presence of genes encoding the CTX-M-1 and CTX-M-2 group of enzymes was confirmed (Table 5). In addition, isolates of *C. freundii* were characterized also by the presence of *bla_CTX-M-8/25_* genes. Resistance to cephalosporin ATBs in 3 isolates of *L. amnigena* was encoded by *bla_CTX-M-1_* genes, while two were also possessed by *bla_CTX-M-2_* and *bla_CTX-M-8/25_* genes. Whereas in the isolate of *C. gillenii* the production of the CTX-M-1 and CTX-M-2 group of enzymes was not encoded, the presence of the *bla_CTX-M-8/25_* group of genes was observed. Among genes encoding CTX-M type of ESBLs, *K. pneumoniae* possessed only by *bla_CTX-M-1_* genes (Table 5). Many studies indicate a higher prevalence of CTX-M-type ESBLs compared to TEM-type enzymes in clinical isolates of *E. coli* [47,49], which was confirmed also in the present work. While the presence of *bla_TEM_* genes was reported only in 9 isolates of *E. coli*, different variants of *bla_CTX-M_* genes were found in 15 isolates. Specifically, from all 19 tested isolates the highest number (10 isolates) was positive for the presence of *bla_CTX-M-8/25_* genes. Conte et al. (2017) also noted a high prevalence of the *bla_CTX-M-8_* gene including to the *bla_CTX-M-8/25_* group of genes in ESBL-producing *E. coli* isolates [35]. CTX-M-8 enzyme was first described in clinical samples in Brazil and subsequently also in environmental samples. Bacteria producing this type of ESBL have been detected only sporadically in hospital settings but have been prevalent among isolates from food-producing animals and chicken meat [35]. The presence of genes encoding the CTX-M-2 group of enzymes was found in nine isolates, while five were positive for the presence of *bla_CTX-M-1_* genes (Table 5).

At the present time, among all the CTX-M-type of ESBLs the most widespread is CTX-M-15, whose production was first described in *E. coli* [22]. In case of all isolates of *C. freundii* as well as in one isolate of *L. amnigena* and *K. variicola.* the group of *bla_CTX-M-1_* genes was specifically represented by this gene (Table 5). Memariani et al. (2015) indicate the coexistence of the *bla_CTX-M-15_* gene with other ESBL-encoding genes such as *bla_TEM_* and *bla_SHV_* or even with genes encoding resistance to other ATBs including *qnr* and *aac(6’)-Ib-cr* genes [22]. The presence of a gene encoding the CTX-M-15 production was always detected, together with *bla_TEM_*, *bla_OXA_* and *bla_CTX-M-2_* genes, highlighted also in the study of Sun et al. (2018) [50]. 

### 3.4. The Ability of ATB-Resistant Isolates to form Biofilm

The production of biofilm was observed in all coliform bacteria isolated from HEs. More than half were strong producers of bacterial biofilm. One third of isolates were strong biofilm producers, whereas 20% were medium producers of biofilm (Table 6). It was assumed that the high proportion of coliform bacteria included among strong and very strong biofilm producers isolated from HEs were derived from patients as well as contaminated catheter surfaces or other devices. Despite efforts to maintain sterility, implantable and prosthetic medical devices can easily become contaminated with bacteria. Bacterial biofilms have been most often identified on urinary catheters, endoscopes, tracheal tubes, or breast implants. According to National Institute of Health, biofilms account for up to 80% of the total number of microbial infections in humans. Major challenges in treating such infections are their difficult diagnosis as well as a high tolerance of pathogens attached in biofilms to antibiotics [51].

## 4. Conclusions

The hospital environment as well as its wastewaters is of great concern in the development and dissemination of antibiotic resistance. Our data show that effluent wastewater from health care facilities such as hospitals in the Czech and Slovak Republic contains a high number of antibiotic resistant coliform bacteria with MDR phenotype. The majority of antibiotic resistant coliform bacteria isolated from Slovak HEs were MDR with confirmed efflux pump overproduction. The alarming fact is that these strains also produce ESBL and possess a combination of two or more antibiotic resistance genes which can be transferred to antibiotic susceptible bacteria in municipal sewerage. Moreover, the majority of isolates are strong biofilm producers, so they can survive, multiply and disseminate antibiotic resistance genes attached to sewerage walls. 

## Figures and Tables

**Table 1:** Characteristics of healthcare facilities.

**Hospital (SR)**	**Number of Beds**	**Equipment Type**	**Recipient**
Psychiatric Clinic in Oščadnica (PC)		outpatient care	River Oščadničanka
National Cancer Institute of St. Elizabeth (NCISE)	198	hospital with outpatient care	Public sewerage network
University Hospital in Ružinov	875	hospital with outpatient care	
University Hospital of Academician Ladislav Dérer	635	hospital with outpatient care	
Children´s Faculty Hospital	397	hospital with outpatient care	
Hospital in Malacky	222	hospital with outpatient care	
**Hospital (CZ)**	Number of beds	Equipment type	Recipient
Policlinic in Rožnov pod Radhoštěm		ambulance	Public sewerage network
Hospital in Vsetín	350	hospital with outpatient care	
Hospital in Valašské Meziříčí (HVM)	190	hospital with outpatient care	
Hospice Citadela	450	palliative and outpatient care	

**Table 2:** Oligonucleotides used in this study.

	Primer	Sequence (5’ to 3’ Direction)	Primer Volume (μL)	Amplicon Size (bp)	Source
**Multiplex** **I**	TEM_fwd	CATTTCCGTGTCGCCCTTATTC	1	800	[21]
	TEM_rev	CGTTCATCCATAGTTGCCTGAC	1		
	SHV_fwd	AGCCGCTTGAGCAAATTAAAC	1	713	
	SHV_rev	ATCCCGCAGATAAATCACCAC	1		
	OXA_fwd	GGCACCAGATTCAACTTTCAAG	1	564	
	OXA_rev	GACCCCAAGTTTCCTGTAAGTG	1		
**Multiplex II**	CTXMGp1_fwd	TTAGGAARTGTGCCGCTGYA ^a^	0.25	688	
	CTXMGp1_rev	CGATATCGTTGGTGGTRCCAT ^a^	0.25		
	CTXMGp2_fwd	CGTTAACGGCACGATGAC	0.25	404	
	CTXMGp2_rev	CGATATCGTTGGTGGTRCCAT ^a^	0.25		
**Single PCR**	CTXM8/25_fwd	AACRCRCAGACGCTCTAC ^a^	0.25	326	
	CTXM8/25_rev	TCGAGCCGGAASGTGTYAT ^a^	0.25		
	CTXM15_fwd	CACACGTGGAATTTAGGGACT	1	995	[22]
	CTXM15_rev	GCCGTCTAAGGCGATAAACA	1		
**Tet Multiplex**	TetA_fwd	GCTACATCCTGCTTGCCTTC	0.5	210	[23]
	TetA_rev	CATAGATCGCCGTGAAGAGC	0.5		
	TetE_fwd	AAACCACATCCTCCATACGC	0.5	278	
	TetE_rev	AAATAGGCCACAACCGTCAG	0.5		

^a^ Y = T or C, R = A or G, S = G or C.

**Table 3:** Number of total and resistant coliform bacteria (*E. coli*) in hospital effluents (HEs).

	CFB (log CFU·mL^−1^)	EC (log CFU·mL^−1^)
**Total**	ND-7.18 ± 0.32	ND-4.49 ± 0.15
**AMP (EU)**	ND-7.17 ± 0.41	ND-4.37 ± 0.31
**GEN (EU)**	ND-6.59 ± 0.18	ND-3.81 ± 0.16
**CIP (EU)**	ND-6.39 ± 0.21	ND-3.69 ± 0.09
**CMP (EU)**	ND-5.41 ± 0.14	ND-2.86 ± 0.06
**AMP (US)**	ND-7.17 ± 0.12	ND-4.35 ± 0.11
**GEN (US)**	ND-6.45 ± 0.22	ND-3.63 ± 0.08
**CIP (US)**	ND-5.79 ± 0.17	ND-3.59 ± 0.14
**CMP (US)**	ND-5.00 ± 0.25	ND-2.62 ± 0.19
**TET (US)**	ND-5.86 ± 0.09	ND-3.73 ± 0.07

CFB—coliform bacteria, EC—*E. coli*, AMP—ampicillin, GEN—gentamicin, CIP—ciprofloxacin, CMP—chloramphenicol, TET—tetracycline, ND—not detected, EU—resistance breakpoints according to the European Committee on Antimicrobial Susceptibility Testing (EUCAST), US—resistance breakpoints according to the Clinical Laboratory Standards Institute (CLSI).

**Table 4:** Susceptibility and multidrug resistant (MDR) phenotype of resistant isolates of coliform bacteria.

		Number of resistant isolates				
		*E. coli*	*Citrobacter* spp.	*L. amnigena*	*E. cloacae*	*Klebsiella* spp.
**Number of isolates**		19	6	6	2	2
**Antibiotics**	AMP	19	INR	6	INR	INR
	AMS	14	-	-	-	2
	CFZ	13	-	-	-	2
	CXM	10	-	-	-	2
	AZT	7	-	-	-	1
	GEN	16	6	2	1	2
	AMK	7	-	-	-	0
	COL	3	-	-	-	1
	T/S	8	-	-	-	2
	CIP	9	5	5	1	2
	CMP	7	5	2	1	1
	TET	14	6	5	2	2
	PIP	17	-	-	-	2
	PIT	6	-	-	-	1
	CTX	9	-	-	-	1
	CAZ	12	6	6	2	1
	CPZ	8	-	-	-	2
	CPS	-	-	-	-	-
	CEP	12	-	-	-	1
	MER	0	0	0	0	0
	ERT	3	-	-	-	0
	TGC	0	-	-	-	0
	NET	14	-	-	-	1
	TOB	15	-	-	-	1
**% MDR**		79	100	100	100	100

AMP-ampicillin, AMS-ampicillin/sulbactam, CFZ-cefazolin, CXM-cefuroxime, AZT-aztreonam, GEN-gentamicin, AMK-amikacin, COL-colistin, T/S-trimethoprim/sulfamethoxazole, CIP-ciprofloxacin, CMP-chloramphenicol, TET-tetracycline, PIP-piperacillin, PIT-piperacillin/tazobactam, CTX-cefotaxime, CAZ-ceftazidime, CPZ-cefoperazone, CPS-cefoperazone/sulbactam, CEP-cefepime, MER-meropenem, ERT-ertapenem, TGC-tigecycline, NET-netilmicin, TOB-tobramycin. INR-intrinsic resistance, MDR-multidrug-resistance.

**Table 5:** Characterization of antibiotic resistant coliform bacteria isolated from HEs.

	Isolate	Phenotype	ESBLs	Efflux Pumps	*tet* Genes
**O1**	*E. coli*	-	TEM,OXA,CTX-M-1	-	-
**O2**	*E. coli*	-	TEM,CTX-M-2,CTX-M-8/25	*-*	*tetA*
**O3**	*E. coli*	ESBL	SHV,CTX-M-2,CTX-M-8/25	*-*	*tetA*
**O4**	*E. coli*	ESBL	SHV,CTX-M-2,CTX-M-8/25	-	-
**O5**	*E. coli*	ESBL	SHV,CTX-M-2	+	-
**O6**	*E. coli*	ESBL	SHV,CTX-M-2,CTX-M-8/25	*+*	*tetA,tetE*
**O7**	*E. coli*	ESBL	SHV,CTX-M-2,CTX-M-8/25	*+*	*tetA,tetE*
**O8**	*E. coli*	ESBL	SHV,CTX-M-2,CTX-M-8/25	*+*	*tetA,tetE*
**O9**	*E. coli*	ESBL	CTX-M-1,CTX-M-2,CTX-M-8/25	*+*	*tetA,tetE*
**O10**	*E. coli*	AmpC	SHV,CTX-M-2,CTX-M-8/25	*+*	*tetA,tetE*
**O11**	*L. amnigena*	ESBL	TEM, SHV,OXA,CTX-M-1,CTX-M-2,CTX-M-15	*+*	*tetA,tetE*
**O12**	*E. cloacae*	ESBL	-	-	-
**O13**	*L. amnigena*	-	-	-	-
**O14**	*E. cloacae*	-	-	-	-
**O15**	*L. amnigena*	-	CTX-M-2,CTX-M-8/25	*+*	*tetA*
**O16**	*L. amnigena*	-	CTX-M-1,CTX-M-2,CTX-M-8/25	*-*	*tetA*
**O17**	*L. amnigena*	ESBL + AmpC	-	-	-
**O18**	*L. amnigena*	ESBL	-	-	-
**P1**	*E. coli*	-	TEM,CTX-M-8/25	-	-
**P2**	*E. coli*	-	TEM,SHV	+	-
**P3**	*E. coli*	-	-	+	-
**P4**	*E. coli*	-	TEM	*+*	*tetA*
**P5**	*E. coli*	-	TEM,CTX-M-8/25	*+*	*tetA*
**P6**	*E. coli*	-	-	*+*	*tetA*
**P7**	*E. coli*	-	TEM,CTX-M-1	*+*	*tetA*
**P8**	*E. coli*	-	TEM,CTX-M-1	*+*	*tetA*
**P9**	*E. coli*	-	TEM,OXA,CTX-M-1	*+*	*tetA*
**P10**	*C. freundii*	ESBL	TEM,OXA,CTX-M-1,CTX-M-2,CTX-M-8/25,CTX-M-15	*+*	*tetA*
**P11**	*C. freundii*	-	TEM,OXA,CTX-M-1,CTX-M-2,CTX-M-8/25,CTX-M-15	*-*	*tetA,tetE*
**P12**	*C. freundii*	ESBL	TEM,OXA,CTX-M-1,CTX-M-2,CTX-M-8/25,CTX-M-15	*-*	*tetA,tetE*
**P13**	*C. freundii*	ESBL	TEM,OXA,CTX-M-1,CTX-M-2,CTX-M-8/25,CTX-M-15	*-*	*tetA,tetE*
**P14**	*C. freundii*	ESBL	TEM,OXA,CTX-M-1,CTX-M-2,CTX-M-8/25,CTX-M-15	*-*	*tetA,tetE*
**P15**	*C. gillenii*	-	TEM,OXA,CTX-M-8/25	-	-
**P16**	*K. variicola*	-	TEM,SHV,OXA,CTX-M-1,CTX-M-2,CTX-M-15	*+*	*tetA,tetE*
**P17**	*K. pneumoniae*	-	TEM,SHV,OXA,CTX-M-1	*-*	*tetA*

O1-O18—isolates from the National Cancer Institute of Saint Elizabeth (NCISE). P1-P17—isolates from the Psychiatric Clinic (PC).

**Table 6:** Ability of antibiotic resistant coliform bacteria isolated from HEs to form biofilm.

Intensity of Biofilm Production	Number of Antibiotic Resistant Isolates				
	*E. coli* (19)	*Citrobacter* spp. (6)	*Lelliottia amnigena* (6)	*E. cloacae* (2)	*Klebsiella* spp. (2)
**low**	0	0	0	0	0
**medium**	7	0	0	0	0
**strong**	9	1	6	2	1
**very strong**	3	5	0	0	1
